# Molecular and serologic analysis of linked blood donor and recipient samples to rule out transfusion-transmitted parvovirus B19 infection

**DOI:** 10.3389/fpubh.2026.1819397

**Published:** 2026-05-12

**Authors:** Maureen J. Miller, Maria Luisa Virata, Hailing Yan, Lilin Zhong, Sreya Tarafdar, Sarah Fowler, Pei Zhang, Harvey J. Alter, Valeria De Giorgi

**Affiliations:** 1 Department of Transfusion Medicine, National Institutes of Health Clinical Center, Bethesda, MD, United States; 2 Plasma Derivatives Branch 2, Division of Plasma Derivatives, Office of Plasma Protein Therapeutics, Office of Therapeutic Proteins, Center for Biologics Evaluation and Research, U.S. Food and Drug Administration, Silver Spring, MD, United States

**Keywords:** blood donors, parvoviral infection, parvovirus, B19, transfusion-transmitted infections, emerging infectious diseases

## Abstract

**Introduction:**

Parvovirus B19 (B19V) has been found in asymptomatic blood donors. This prospective study tested linked blood donor–recipient samples from patients transfused at the National Institutes of Health (NIH) and affiliated hospitals for the presence of B19V DNA and anti-B19 immunoglobulin G (IgG) antibodies to assess the risk of B19V transmission by transfusion.

**Materials and methods:**

Post-transfusion recipient plasma samples collected during 2002–2020 were tested for B19V DNA at 1, 2, 4, and/or 8 weeks by nested polymerase chain reaction (PCR), and for anti-B19 IgG at 12 and ≥24 weeks by enzyme immunoassay (EIA). To confirm B19V DNA-positive recipients, pre-transfusion and linked donation samples were tested for B19V DNA, anti-B19 IgG, and/or IgM. Tested samples included 1,352 subjects tested for DNA and anti-B19V IgG, or both.

**Results:**

No new transfusion-transmitted B19V infections were identified. Only one confirmed transfusion-transmitted infection (TTI) was identified overall. A total of 19 of 1,314 subjects tested for B19V DNA (1.4%, 95% confidence interval [CI]: 0.014 [0.009, 0.021]) had a positive result in their post-transfusion sample. A total of 876 of 1,290 tested subjects were positive for anti-B19V IgG antibody (68%, 95% CI: 0.679 [0.65, 0.70]). Antibody detection fluctuated among some recipients from 12 to 24 weeks.

**Discussion:**

The B19V DNA-positive rate in recipients was very low. All B19V DNA-positive recipients had pre-existing antibodies, indicating prior exposure. Although these data may not provide enough evidence to consider revising current B19V testing strategies, other pooled plasma testing data from around the world suggest that more widespread B19V monitoring of blood products could be indicated to prevent B19V infection in immunosuppressed or otherwise susceptible recipients (e.g., individuals with chronic hematological diseases, pregnant women less than 20 weeks gestation).

## Introduction

Parvovirus B19 (B19V) (*Parvoviridae* family, subfamily *Parvovirinae*, genus *Erythroparvovirus*) (1), a small, non-enveloped, single-stranded DNA virus, can be transmitted to humans via respiratory droplets, vertical maternal-fetal transmission, or hematogenous transmission by transfusion or transplant. The majority of the clinically relevant erythropoietic progenitor cells can be infected (2). Clinical presentations of B19V in humans can vary from an asymptomatic infection, a mild rash, or red blood cell aplasia that can exacerbate other anemias or cause fetal loss (3). The risks associated with B19V infection are not insubstantial, even in healthy adults. Although the overall risk of B19V infection in the general population of Europe (where outbreaks occurred in 2024) is deemed to be low, prior immunity to B19V determines the probability of infection, with secondary transmission in households reaching 50%. Such transmission could potentially affect vulnerable groups such as pregnant women at fewer than 20 weeks of gestation (low to moderate risk), patients with chronic hematological diseases (moderate risk), or immunosuppressed individuals, including transplant patients (moderate risk) (4). Given that B19V can be spread hematogenously and cause severe clinical outcomes, the potential for B19V transmission via transfusion of blood and plasma products is an important consideration in infectious disease screening of the blood supply in any geographic location.

Quantifying the true burden of B19V in the blood supply has proved challenging. The highest titers of B19V (up to 1E+14 IU/mL) are typically recorded about 1 week after infection, then persist for another 5 days before IgM antibody formation and viremia decline 10–14 days post-infection. That said, low-level DNA (<1E+3 IU/mL) has been observed in blood for up to 6 months, even in people with detectable IgG (5). Unlike other transfusion-transmissible infectious pathogens commonly tested in the United States (6, 7), B19V is resistant to standard pathogen reduction methods in both fractionation and amotosalen-ultraviolet A light (a light treatment); for example, high B19 viral titers were measured in several platelet concentrates after they underwent pathogen reduction, suggesting that it is hard to reduce B19V infectivity in these products (8, 9). B19V transmission via blood components or plasma-derived products is rare (10, 11); in fact, we previously reported only one case of B19V transmission due to transfusion of infected donor red blood cell concentrates (12). Given its rarity, the true risk of B19V transmission via transfusion is uncertain. Blood products in the US that are not destined for fractionation are not routinely screened for B19V. Without such screening, it has been difficult to estimate the true prevalence of B19V in healthy blood donors or the transmission risk to blood recipients (13).

Prospective monitoring of linked donor–recipient interactions can track potentially emerging pathogens, such as B19V, over time. We report herein updated parvovirus B19 viral DNA and antibody data from a prospectively studied cohort of US blood donors and recipients (14). Preliminary results of this study indicated a low risk of parvovirus B19 transmission in the cohort during 2002–2010 (12). This article presents the final results of B19V testing for the entire A Prospective Study of Transfusion-Transmitted Infections (Transfusion-Related Infections, Prospectively Studied) (TRIPS) study cohort, reported in full, including the previously published study period 2002–2010, with new results for the study period 2010–2020. The objective of this study was to monitor the transfusion risk of B19V by testing linked blood donor–recipient samples taken from participants at three academic hospital centers in the metropolitan Washington, D. C. area of the US for the presence of B19V DNA and anti-B19 IgM and IgG antibodies to investigate the risk of B19V transmission by blood transfusion.

## Materials and methods

### Institutional review board/human subjects research

These data were collected via A Prospective Study of Transfusion-Transmitted Infections (Transfusion-Related Infections, Prospectively Studied) (TRIPS), an observational study approved as human subjects research by the National Institutes of Health Clinical Center (NIH CC) Institutional Review Board (NIH CC IRB Protocol #01-CC-0231).

### Data source (study cohort)

Biospecimen samples were collected from 1,771 volunteer participants at 3 metropolitan Washington, D. C. area hospitals (NIH Clinical Center, Suburban Hospital, Children’s National Medical Center) from January 2002 to March 2020. Patients were eligible for the study protocol if they were at one of the three hospitals for a blood transfusion, usually as part of participation in surgical protocols (cardiac surgery or transplant surgery for cancer care), care for immunocompromising conditions (e.g., cancer, blood disorders, infectious diseases, or congenital disorders), or care for chronic hematological diseases that would be deemed very low to moderate risk for B19V infection according to a risk assessment system proposed by the European Centre of Disease Prevention and Control in 2024 after the completion of the study (4). In general, people transfused at these hospitals were eligible for the study if: (1) they had not been transfused in the 6 weeks preceding the index transfusion (meaning frequently transfused recipients were not enrolled more than once); (2) they were expected to remain in the continental US for at least 6 months after the index transfusion; and (3) if they consented, a pre-sample was obtained, and they received a transfusion during their hospital stay. As many eligible participants as the study staff were resourced to enroll were included in the study. There were no explicit exclusion criteria for transfusion recipients. Donors linked to recipients were excluded if they were deferred from donating blood according to US Food and Drug Administration (FDA) guidelines.

### Testing

#### General approach to testing

Whole blood or plasma samples were tested for B19V markers. Serial post-transfusion recipient plasma samples collected at 1, 2, 4, and/or 8 weeks were tested for B19V DNA by nested polymerase chain reaction (PCR), while recipient plasma samples collected at 12 and 24 weeks post-transfusion (or at end of study, EOS) were tested for anti-B19V IgG by an enzyme immunoassay (EIA).

**Figure 1:**
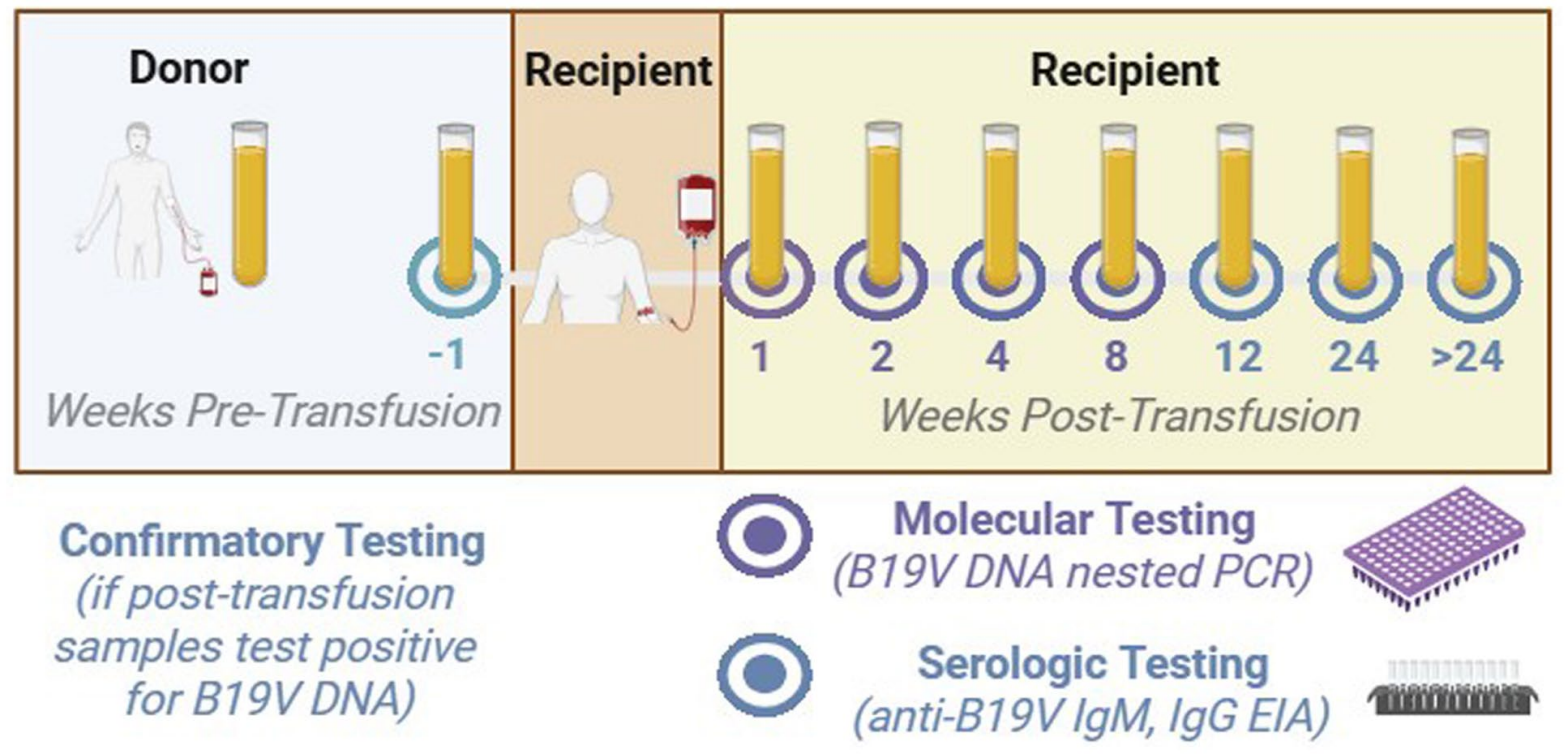
Overview of blood testing in the study protocol. This study monitored and tested recipients of blood product transfusions (*center*) for multiple infectious diseases in the pre- and post-transfusion periods and linked recipients’ pre- and post-transfusion test results (*upper right*) to infectious diseases tests results for the donors of the blood products they received (*upper left*). Post-transfusion recipient plasma samples were tested for B19V DNA at 1, 2, 4, and/or 8 weeks by nested PCR (*lower right*), and for anti-B19V IgG at 12 and 24 weeks (or more) by EIA (*lower right*). To confirm B19V DNA-positive recipients, pre-transfusion and linked donation samples were tested for B19V DNA, anti-B19V IgG, and/or IgM (*lower left*). If positive, linked donor and recipient sequences were compared. Serum and plasma were tested for B19V. Except where specifically required, the pre-transfusion sample was tested only if the post-transfusion sample tested positive. Image created in BioRender.

To further investigate B19V DNA-positive recipients (i.e., containing ≥ 20 IU/mL B19V DNA), pre-transfusion and linked donation samples were tested for B19V DNA, anti-B19V IgG, and/or IgM (Figure 1). If the pre-transfusion sample tested negative for B19V DNA, while a linked donor sample tested positive, then the DNA sequences of the linked donor(s) and recipient were compared. Antibody seroconversion was confirmed if a pre-transfusion recipient sample tested repeatedly negative for anti-B19V and at least two post-transfusion samples tested positive. In cases without data for all specified timepoints, we considered data from at least one early post-transfusion sample (1, 2, or 4 weeks) and at least one late timepoint (i.e., 12 or 24 weeks, or end of study [EOS]) to be an acceptable indicator of parvovirus B19 infection. That is, donors were tested only when recipient samples were positive.

#### Defining a transfusion-transmitted infection

A transfusion-transmitted infection (TTI) was defined as follows: Subjects who had study samples obtained from multiple bleeding dates before and after their transfusion at intervals deemed sufficient to routinely detect molecular evidence of infection. The vast majority of antibody seroconversions within 3 months of the index transfusion were analyzed. If B19V was found in a post-transfusion sample, a pre-transfusion sample was tested to determine whether the infection preceded or followed the transfusion episode. If the infection was found to be temporally related to the transfusion, donor samples were tested to link infection in the donor and recipient and provide evidence for transmission of the identified agent by transfusion. Where there was molecular and serologic evidence of transmission of the identified agent between donor and recipient, viral genome sequences were recovered from the donor and recipient and compared. A matching sequence was considered a confirmed transfusion-transmitted infection.

#### Molecular testing

Whole blood or plasma samples collected from recipients at 4 and 8 weeks post-transfusion, and, later in the study, also collected at 1 and 2 weeks post-transfusion, were tested for the presence of B19V DNA by nested polymerase chain reaction (PCR). DNA was extracted from 0.2 mL (and, later in the study, 0.1 mL) of plasma using the QIAamp^®^ MinElute^™^ Virus Spin isolation kit (Qiagen, Inc., Valencia, CA, USA) and recovered in 0.1 mL of elution buffer, according to the manufacturer’s instructions. The following in-house B19V nested PCR procedure was used: A 25-μL aliquot of undiluted DNA extract was added to a 25-μL master mix such that the final reaction mixture for the first-round amplification contained 10-mM Tris–HCl pH 8.3, 50-mM KCl, 4.5-mM MgCl_2_, 0.001% (wt/vol) gelatin, 0.025 units of AmpliTaq DNA polymerase (Thermo Fisher Scientific, Carlsbad, CA, USA), 0.1 mM for each of the 4 dNTPs, and 0.2 μM each of the first set of B19V oligonucleotide primers. Over the course of the study, we used two PCR primer sets: one we described in our 2010 *Transfusion* paper (12), used from 2002 to 2009, and another used beginning in 2009 that also covered the VP1/VP2 region of the B19V Au sequence. The redesigned PCR primer set generated a larger amplicon, resulting in a more intense, readily detectable band on the agarose gel. Results from either primer set were deemed to be equivalent. Notably, 1 μL of the first-round amplicon was then transferred to the 49-μL second-round amplification master mix, which contained the second set of B19V primers (0.2 μM each) along with the same enzyme and buffer constituents as in the first round. Nested PCR B19V primer sequences were derived from the VP1/VP2 region of the human parvovirus B19 (Au isolate) genome (15); nucleotide numbering was based on the published B19V Au sequence (GenBank accession number: M13178). The first set of (external) primers was forward primer: 5′-ACCACCCCCATGCCTTATC-3′ (nucleotides 2,709–2,727) and reverse primer: 5′-CTGCACCAGTGCTGGCTTC-3′ (nucleotides 3,164–3,146), while the second set of (internal) primers were forward primer: 5′-ACAAGCCTGGGCAAGTTAGC-3′ (nucleotides 2,790–2,809) and reverse primer: 5′-GCTGGCTTCTGCAGAR*TTAAC-3′ (nucleotides 3,154–3,134). The inclusion of the degenerate base R*(A/G) was intended to account for potential allelic variation observed in genotype 1 and genotype 2 sequences, to maintain amplification efficiency across both genotypes. This PCR assay can detect B19V genotypes 1 and 2 but not genotype 3.

DNA amplification was carried out in a thermal cycler (Applied Biosystems Veriti^™^ model, Thermo Fisher Scientific, Carlsbad, CA, USA) with the following programmed settings: For the first-round PCR, an initial heating at 94 °C for 3 min was followed by 30 cycles of 94 °C for 30 s, 55 °C for 30 s, and 72 °C for 90 s. For the second-round PCR, 30 cycles of 94 °C for 15 s, 55 °C for 30 s, and 72 °C for 50 s were performed. Second-round PCR products (2 μL per test sample or control) were analyzed by electrophoresis on a 1% agarose gel pre-coated with a fluorescent nucleic acid gel stain (GelRed Agarose LE, Biotium, Fremont, CA, USA). Stained DNA bands on the gel were visualized using a fluorescence/chemiluminescence imaging system (G: BOX mini 6 model, Syngene, Frederick, MD, USA). Samples with one visible amplicon band of 364 bp (nucleotides 2,790–3,154) on the gel were considered positive for B19V DNA. The first World Health Organization (WHO) International Standard for Parvovirus B19 DNA for Nucleic Acid Amplification (NAT) Assay (NIBSC code 99/800, 10^6^ IU/mL, when reconstituted) was diluted 10^3^-fold and served as the positive control both for extraction and quantification. DNA extracts of the B19V DNA reference standard were diluted further (10^0.5^, 10^1.0^, and 10^2.0^) as per limiting dilution analysis; duplicates of each dilution were included in each nested PCR assay and run on the same agarose gel to help estimate the amount of B19V DNA in DNA-positive recipient samples. Normal human plasma was used as the negative control.

A post-transfusion sample with a positive result after the initial nested PCR assay was confirmed to be a true B19V DNA-positive only if a second separate 0.1 mL sample aliquot also tested positive. Once a post-transfusion sample was confirmed positive after re-testing, the pre-transfusion sample was requested from the repository, and a 0.1-mL aliquot was similarly tested by nested PCR. If the pre-transfusion sample tested negative for B19 DNA, a B19V transmission by transfusion was considered possible, but only confirmed if a linked donor’s plasma or whole blood sample also tested positive for B19V DNA.

#### Serologic testing

A majority of plasma samples from recipients were tested by enzyme immunoassay (EIA) at a molecular virology laboratory at the FDA, while 14 samples were sent to Mayo Clinic Laboratories (Rochester, MN, USA) for serologic testing (16).

The 12- and 24-week post-transfusion plasma samples were tested for anti-B19V IgG using an anti-B19V IgG EIA kit (Biotrin International Ltd., Dublin, Ireland) according to the manufacturer’s protocol. The assay is intended for the qualitative detection of IgG or IgM, but an index value is used to help interpret the results. The index value was obtained by dividing the absorbance value of the test sample by the cutoff value, which is, as per enzyme immunoassay (EIA) test kit instructions, computed by multiplying the calibrator’s mean absorbance by the lot-specific constant. An index value <0.9 or >1.1 indicates sample negativity or positivity, respectively. An index value in the range 0.9–1.1 indicates equivocality. Ten microliter aliquots of plasma samples were assayed alongside kit calibrator and controls on the same 96-well microtiter plate coated with B19V recombinant VP2 capsid protein. Inter-assay reproducibility of the Biotrin EIA assay can range from 5.9 to 23%. For confirmed B19V DNA-positive recipients, all their serial plasma samples (pre-transfusion, earlier post-transfusion time points) and linked donation samples were also tested for anti-B19V IgM using an anti-B19V IgM EIA kit (Biotrin).

#### Data analysis

Data analysis was performed using Microsoft Excel software (Microsoft Corporation, Redmond, WA, USA) and GraphPad Prism version 11.0.0 (GraphPad Software, Boston, MA, USA).

## Results

### Study subjects

**Figure 2:**
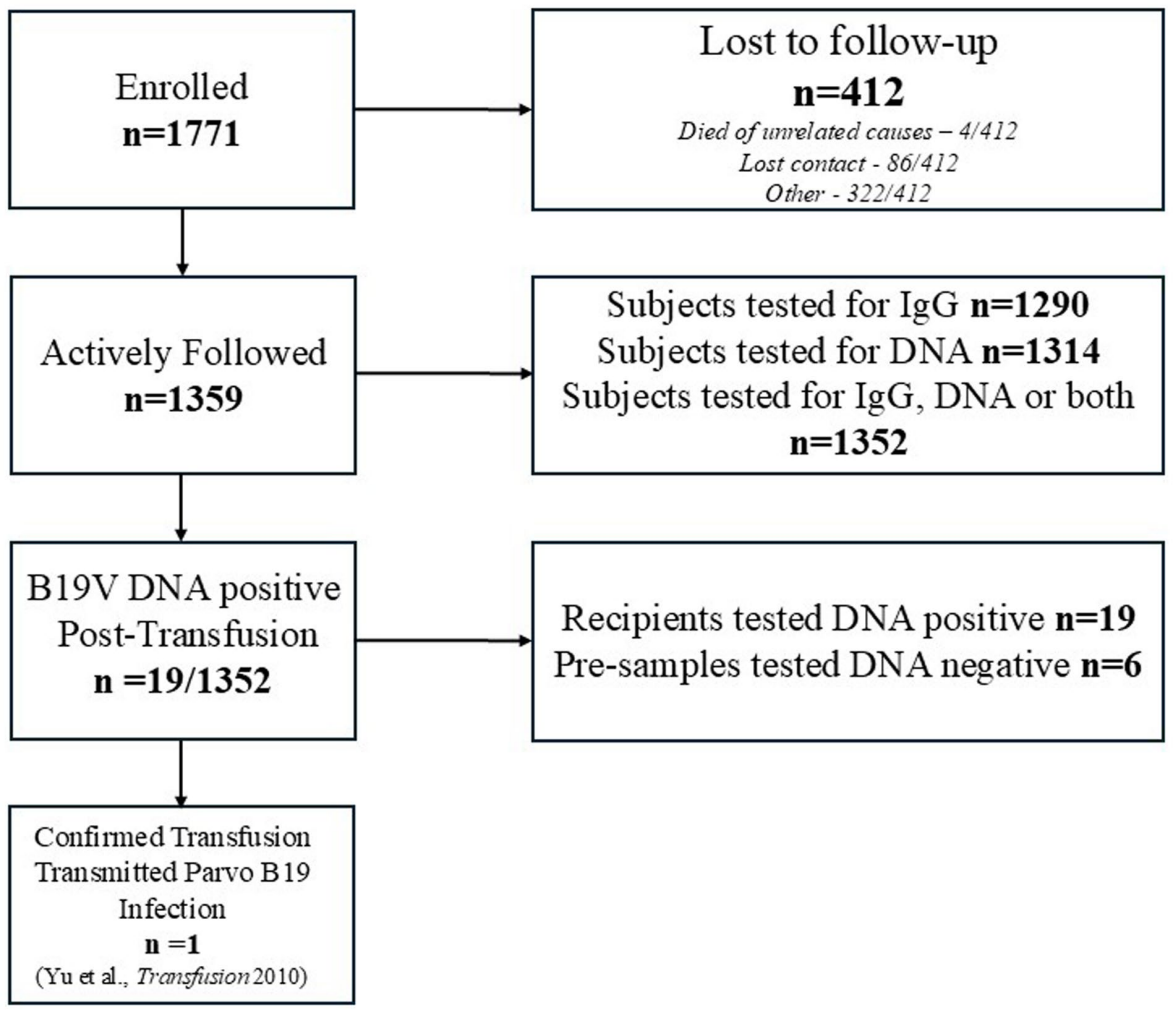
Flowchart of the study inclusion criteria. A total of 1771 blood recipients were enrolled in the epidemiologic study, of whom 1,359 completed the study, and 412 were lost to follow-up. Of the 1,359 recipients who completed the study, 1,352 were tested for either B19V DNA, anti-B19 IgG, or both for at least one timepoint. Only 1 of these 1,352 recipients had a transfusion-transmitted B19V infection.

A total of 1,771 blood recipients were enrolled in the epidemiologic study (Figure 2). Of 1,771 study subjects, 1,359 completed the study and 412 were lost to follow-up (199 [48%] too ill to complete study, 86 [21%] lost contact, 44 [11%] could not get follow-up laboratories drawn, 33 [8%] non-compliant with follow-up laboratory draw schedule, 18 [4.3%] ended participation after not completing laboratories due to the coronavirus disease 2019 [COVID-19] pandemic, 16 [3.9%] requested to leave the study, 6 [1.4%] gave insufficient sample quantity for testing, 6 [1.4%] left study for unknown reasons). Four patients died of unrelated causes before submitting all laboratory samples. The mean age of subjects was 40.9 years (range: 0–89 years). Out of the 1,359 subjects who completed the study, 789 subjects were male (58%) and 570 (42%) were female; 828 participants identified as non-Hispanic White (60.9%), 348 Black or African–American (25.6%), 98 Hispanic or Latino (7.2%), 35 Asian (2.6%), 4 as more than one race (0.3%), and 1 American Indian/Alaska Native (0.1%); race/ethnicity was unknown or reported as other for 45 (3.3%). Clinical characteristics of the 1,359 subjects who completed the study were as follows: 415 participants (30.5%) had general population (very low) risk for B19V infection (e.g., cardiac surgery), 835 (61.4%) were potentially immunosuppressed individuals with a primary or secondary diagnosis suggestive of immunosuppression (e.g., under immunosuppressants, immunosuppression due to HIV infection, cancers including malignant blood cancers, transplantation, etc.) (moderate risk for B19V infection), 86 (6.3%) had chronic hematological diseases (including patients, some frequently transfused, with hemolytic anemias, e.g., sickle cell disease, thalassemia, hereditary spherocytosis, etc.) (moderate risk for B19V infection), and 23 (1.7%) were at unknown risk for B19V infection (e.g., missing information; individuals with infections or precancers without context on their progression to diseases causing immunosuppression). Pregnancy status was unavailable for these subjects; the risk of B19V infection for pregnant women less than 20 weeks is low-to-moderate (4).

### Testing results

A total of 1,352 (1,352/1,359 [99.5%]) subjects who completed the study were tested for either B19V DNA, anti-B19V IgG, or both for at least one timepoint, so we use the number of subjects tested in the denominators to describe percent test positivity. Specifically, a total of 2,197 samples from 1,314 blood transfusion recipients (1,314/1,352 [97.2%] subjects) were tested for B19V DNA, and a total of 2,301 samples from 1,290 blood transfusion recipients (1,290/1,352 [95.4%] subjects) were tested for anti-B19V IgG (Table 1).

**Table 1:** Molecular and serologic testing summary data for parvovirus B19 in transfused recipients.

	B19V DNA Molecular Testing		Anti-B19V IgG Immunoassay	
	(2,197 total specimens tested*)		(2,301 total specimens tested*)	
	Number of subjects	Percentage of subjects	Number of subjects	Percentage of subjects
Total number of subjects tested*	1,352	99.5%	1,352	99.5%
Number of subjects tested for DNA and anti-B19V only	1,314	97.2%	1,290	95.4%
Positive^†^	19	1.4% (0.014 [0.009, 0.021]) (95% confidence interval)	876	67.9% (0.679 [0.65, 0.70]) (95% confidence interval)

*A total of 1,359 subjects completed the study; 1,352 subjects were tested for B19V DNA, anti-B19V IgG, or both (99.5%) at one or more study timepoints. This table uses the number of study subjects tested as the denominator for test positivity.

†For the two proportions measuring molecular and serologic test positivity, 95% confidence intervals were calculated using the number of subjects tested as the sample sizes (i.e., 1,314 participants for molecular testing, 1,290 participants for serologic testing) and α = 0.05.

#### Serologic testing results

A total of 876 of 1,290 recipients (0.679 ± 95% confidence interval [0.65, 0.70]) were positive for anti-B19V IgG antibody (i.e., 67.9% of all subjects who were tested for anti-B19V IgG tested positive for anti-B19V IgG) (Table 1). Typical antibody kinetics in immunocompetent individuals are: IgM appears in 10–14 days post-infection and can persist for 2–4 months (occasionally up to 12 months). IgG appears 15–21 days post-infection, peaks around 30 days, and can persist for life, indicating immunity. For immunosuppressed recipients, IgM and IgG responses may be masked or atypical.

For some recipients, EIA results fluctuated from positive (index >1.1) to negative (index < 0.9) or from negative to positive from 12 to 24 weeks or more post-transfusion (or at EOS). Serology testing results by timepoint for participants with fluctuating test results (32 “seroconverting” from positive to negative and 6 “seroconverting” from negative to positive).

#### Molecular testing results

A total of 19 of the 1,314 (0.014 ± 95% confidence interval [0.009, 0.021], i.e., 1.4%) recipients were found to be B19V DNA positive in their early post-transfusion specimens. Stratified by clinical risk for B19V infection, 2 of these 19 recipients had the risk of the general population for B19V infection (very low risk for B19V infection) (10.5%), 11 were potentially immunosuppressed (moderate risk for B19V infection) (57.9%), and 6 had chronic hematological diseases (moderate risk for B19V infection) (31.6%). The difference in the proportions of participants at moderate risk versus other risk groups in these 19 participants versus the remaining DNA-negative participants was statistically significant (two-sided Fisher’s exact test, *p* = 0.047).

#### Analysis of recipients who tested positive for B19V DNA after transfusion

After performing molecular and serologic testing on pre- and post-transfusion samples from the study cohort (*n* = 1,352), we identified 5 new recipients not reported in our previous manuscript who were positive for B19V DNA at one or more timepoints post-transfusion (Table 2). Two of the 5 recipients tested DNA-negative in their pre-transfusion samples, and 3 of 5 had pre-transfusion samples positive for B19V DNA. Of those DNA-negative pre-transfusion, there was insufficient linked donor sampling to perform molecular testing to prove or disprove transfusion-related transmission.

**Table 2:** Analysis of 19 recipients positive for parvovirus B19 DNA after transfusion.

Recipient number *(Clinical Diagnosis)*	B19V Risk^††^	Before transfusion (pre-sample)			After transfusion*,^†^		
		B19V DNA (IU/mL)^†^	B19V antibodies^‡^		B19V DNA (IU/mL)^†^	B19V antibodies^‡^	
			IgG	IgM		IgG	IgM
Recipient 1 *(sickle cell disease (hemoglobin SS [HbSS]) with crisis, pediatric)*	Moderate	63	Positive	Negative	20	Positive	Negative
Recipient 2 *(congenital heart disease (coarctation of the aorta), pediatric)*	Moderate	20	Positive	Negative	40	Positive	Negative
Recipient 3 *(HIV, adult)*	Moderate	20	Positive	Negative	58	Positive	Negative
Recipient 4 *(hypoglycemia, adult)*	Very low	632	Positive	Positive	1.40E+03	Positive	Negative
Recipient 5 *(congenital heart disease (ventricular septal defect), pediatric)*	Moderate	Negative	Negative	Negative	200	Negative	Negative
Recipient 6 *(renal cell carcinoma, adult)*	Moderate	60	Positive	Negative	40	Positive	Negative
Recipient 7 *(sickle cell disease (HbSS) with crisis, pediatric)*	Moderate	>2E+10	Equivocal	Positive	1.40E+04	Positive	Positive
Recipient 8 *(sickle cell disease (HbSS) with crisis, pediatric)*	Moderate	6.30E+06	Positive	Positive	6.30E+03	Positive	Positive
Recipient 9 *(sickle cell disease (HbSS) with crisis, pediatric)*	Moderate	2.00E+07	Positive	Positive	630	Positive	Positive
Recipient 10 *(low-grade adenocarcinoma of the appendix with peritoneal carcinomatosis, adult)*	Moderate	Negative	Negative	Negative	20**	Positive	Negative
Recipient 11 *(prostate cancer, adult)*	Moderate	Negative	Positive	Negative	630	Positive	Negative
Recipient 12 *(metastatic renal cell carcinoma, adult)*	Moderate	63^§^	Negative	Negative	200	Positive	Negative
Recipient 13 *(metastatic ocular melanoma, adult)*	Moderate	40^§^	Positive	Negative	63^§^	Positive	Negative
Recipient 14 *(congenital heart disease (ventricular septal defect), pediatric)*	Moderate	Negative	Positive	Negative	20	Positive	Positive
**Recipient 15** *(sickle cell disease (HbSS) with crisis, pediatric)*	Moderate	**1.3E+06** ^**§**^	**Positive**	**Positive**	**280**	**Positive**	**Negative**
**Recipient 16** *(thymoma or thymic carcinoma, adult)*	Moderate	**63** ^**§**^	**Positive**	**Negative**	**75.9**	**Positive**	**Negative**
**Recipient 17** *(sickle cell disease (HbSS) with crisis, pediatric)*	Moderate	**1.67E+12**	**Negative**	**Negative**	**3.60E+03**	**Positive**	**Negative**
**Recipient 18** *(coronary artery bypass graft surgery, adult)*	Very low	**Negative**	**Positive**	**Negative** ^**¶**^	**20**	**Positive**	**Negative¶**
**Recipient 19** *(aortic valve replacement surgery for bicuspid valve, adult)*	Very low	**Negative**	**Positive**	**Negative** ^**¶**^	**438.4**	**Positive**	**Negative¶**

*Post-transfusion samples were tested first. Only 19 recipients tested positive; hence, their pre-transfusion samples were obtained from the repository and tested for all B19V markers. All other recipients whose post-transfusion samples were negative for B19V DNA (i.e., <20 IU/mL) were not further investigated. Results for Recipients 15–19 (in bold) are reported for the first time; results for Recipients 1–14 were previously reported (12). This table reproduces the results from the previous manuscript alongside the new results, but some recipients (Recipients 1–14) are identified by different numbers than those used in the previous publication about this cohort (12) to align with updated study results and records after publication. Recipients 1–3 are the same in this manuscript and in the previous publication. Recipient 4 in this manuscript corresponds to Recipient 8 in the previous publication. Recipient 5 in this manuscript corresponds to Recipient 13 in the previous publication. Recipient 6 in this manuscript corresponds to Recipient 4 in the previous publication. Recipient 7 in this manuscript corresponds to Recipient 11 in the previous publication. Recipient 8 in this manuscript corresponds to Recipient 9 in the previous publication. Recipient 9 in this manuscript corresponds to Recipient 10 in the previous publication. Recipient 10 in this publication corresponds to Recipient 14 in the previous publication (with updated B19V DNA but still reflecting the one documented transfusion-transmitted B19V infection). Recipient 11 in this manuscript corresponds to Recipient 5 in the previous publication. Recipient 12 in this manuscript corresponds to Recipient 12 in the previous publication. Recipient 13 in this manuscript corresponds to Recipient 6 in the previous publication. Recipient 14 in this manuscript corresponds to Recipient 7 in the previous manuscript. The reported tiers in IU/mL reflect the geometric mean of all tests performed.

^†^B19V DNA levels listed were determined from the first available samples collected after transfusion, mostly at 4 weeks, except at 2 weeks for Recipients 18 and 19 and at 8 weeks for Recipients 15 and 17.

^§^Post-transfusion samples for several recipients had discrepant testing and re-testing results.

^‡^A designation of positive or negative in this table refers to the results obtained for testing both 12- and 24-week (or end of study (EOS)) samples with the following exceptions: anti-B19V testing was performed only on the 12-week sample for Recipients 9, 11, and 12; only on the 24-week (or EOS) sample for Recipients 5 and 13; and only on the 4-week sample for Recipient 8. Results for Recipient 16 are from samples taken at 16 weeks and EOS, with no testing recorded for 12 weeks.

^¶^The sample was tested at Mayo Clinic Laboratories and not the US Food and Drug Administration, as all other samples were.

^**^This patient was determined to have transfusion-associated parvovirus B19 (genotype 1) transmission (12).

^††^Risk associated with parvovirus B19V infection based on probability of infection and its impact, according to categories proposed by the European Centre for Disease Prevention and Control in the year 2024. In brief, risk is very low in the general population and moderate in immunosuppressed people (e.g., under immunosuppressants, immunosuppression due to HIV infection, cancer, transplantation, etc.) or people with chronic hematological diseases frequently transfused, including patients with hemolytic anemias, e.g., sickle cell disease (sickle cell disease, thalassemia, hereditary spherocytosis, etc.). The risk for pregnant women less than 20 weeks is low to moderate; information on pregnancy status was unavailable for this cohort (4). Individuals were classified as potentially immunosuppressed if they had a primary or secondary diagnosis suggestive of immunosuppression as defined above. All others whose risk group could not be classified were assigned an unknown risk for B19V infection (e.g., missing information or context regarding progression to diseases that increase their risk of B19V infection, such as chronic infections or precancers).

## Discussion

The results of this large, multicenter prospective study of linked blood donors and recipients showed a very low risk B19V transmission by blood product transfusion. Among the 1,314 study participants who received molecular testing, the B19V DNA-positive rate remained low (1.4%) throughout the entire study period (2002–2020). The 19 individuals who were B19V DNA-positive had clinical diagnoses suggestive of an increased risk of B19V infection compared to the overall cohort. The low rate of B19V infection in these samples suggests that there may be a low overall risk of transfusion-transmitted infection by B19V over time. In our 2010 publication in *Transfusion* (12), we reported this single documented TTI of B19V infection in the study cohort. In brief, in 2005, a 35-year-old white female with low-grade adenocarcinoma of the appendix with peritoneal carcinomatosis who had no risk factors for TTIs, no children, nor prior history of blood transfusion became infected with B19V after receiving irradiated, leukoreduced red cell concentrates from 6 donors as part of her surgical protocol. Molecular and serologic testing of samples from the 6 linked donors was performed. Of the six donors, one donor’s plasma sample was not available for testing, one donor was negative for all markers, three donors were anti-B19V IgG-positive, and one donor was highly positive for B19 DNA (5 × 10^9^ IU/mL). The recipient’s 2-, 4-, and 8-week post-transfusion plasma samples were all B19 DNA-positive. Both anti-B19V IgM and IgG became positive in 8 weeks. IgM became negative in 24 weeks (end of study). Sequencing and phylogenetic analysis of the VP1-unique (VP1u) region confirmed a transfusion-associated B19 (genotype 1) transmission in this seronegative recipient. Sequences (710 bps, i.e., 650 bps of VP1u + 50 bps of N-terminal VP2) obtained from the recipient’s plasma samples (both 2- and 8-week samples) were identical to the B19 DNA-positive donor’s plasma and whole blood samples. In contrast, the sequence obtained from the WHO B19 standard (positive control) had 4 nucleotide variations within the VP1u region.

We speculate that all five previously unreported study subjects who tested positive for B19V were infected with B19V prior to transfusion, either recently or remotely. For Recipient #15, both pre-transfusion and 8-week samples were positive for B19V DNA, anti-B19V IgM, and anti-B19V IgG. Both 12-week and EOS samples remained positive for B19 DNA and anti-B19V IgG, possibly due to the presence of IgG antibody–virus complexes. One linked donor sample was anti-B19V IgG-positive but negative for both B19V DNA and IgM. The second linked donor sample was not available. Based on this information, we hypothesize that the recipient was recently infected with B19V prior to transfusion. For Recipient #16, pre-transfusion samples and 3 post-transfusion samples (4-, 8-, and 16-week) contained low levels of B19V DNA and were all positive for anti-B19 IgG, probably due to IgG antibody–virus complexes. All anti-B19V IgM testing was negative. No linked donor sample data for this recipient were available. We hypothesize that the recipient was already infected with B19V prior to transfusion. Recipient #17 had extremely high levels of B19V DNA (1.67E+12 IU/mL) in their pre-transfusion sample and was also B19V DNA-positive at 8 weeks (with no samples from 4 or 12 weeks post-transfusion for comparison). They were also both anti-B19V IgM- and anti-B19V IgG-positive at 8 weeks. By the end of the study, the recipient was anti-B19V IgM-negative but still anti-B19V IgG-positive (index 5.03). No linked donor sample data for this recipient were available. We hypothesize that the recipient was already infected with B19V prior to transfusion in the seronegative window period of infection. Recipient #18 was highly anti-B19V IgG-positive from a previous infection, as indicated by their pre-transfusion sample (index 6.69); no anti-B19V IgM results were available. They then had a low titer of B19V DNA at 2 weeks (20 IU/mL), possibly from antibody–virus complexes, but no other post-transfusion samples were available. All linked donor samples were negative for B19V DNA. Some linked donor samples were anti-B19V IgG-positive. Based on this information, we cannot definitively rule out a transfusion-transmitted infection, given the 2-week sample that was positive for B19V DNA. Still, we hypothesize that the recipient was seropositive due to a prior infection. Recipient #19 was already highly anti-B19V IgG-positive from a previous infection. Despite the presence of anti-B19V IgG, the recipient appeared to be transiently infected, as B19 DNA was detected at 1 week and 2 weeks (with a higher titer at 2 weeks). Perhaps these were antibody–virus complexes. All linked donor samples tested negative for B19V DNA and were anti-B19V IgG-positive. We hypothesize that the recipient was already infected with B19V prior to transfusion, from a previous infection, and then transiently infected with B19V DNA during this study period. The observation of “transient viremia” in previously exposed individuals may be due to the fluctuation of viral DNA levels, which can occur during the course of infection, from undetectable levels below the assay Limit of Detection (LOD) in one sampling timepoint to levels above the assay LOD in the next sampling time point and back down to undetectable levels in subsequent sampling timepoints. However, assay variability could not be excluded. In summary, some transfusion episodes were indeterminate, in that transfusion transmission cannot be proved or disproved for these recipients whose post-transfusion DNA positivity could not be fully interpreted because their pre-transfusion specimen was DNA-negative, with insufficient or unavailable linked donor samples for context (such as the 6 cases of negative to positive seroconversion). The possibility of transient DNAemia in previously exposed individuals and the role of host immune status, especially among immunosuppressed patients, cannot be discounted, as these participants may have masked, atypical immune responses or no response at all.

These results are compatible with published results from epidemiologic studies of B19V transmission conducted over the last 20 years. By comparison, in 2009, another NIH-funded linked donor–recipient study, the Recipient Epidemiology Donor Study (REDS-II) study, showed that 112 B19V DNA–positive components from 105 donors (a total of 12,529 tested donations) that were transfused into a population of surgical patients with a pre-transfusion B19V IgG seroprevalence of 78% showed little additional seroconversion. In that study, the researchers found no transmission to 24 susceptible recipients from the transfusion of components with B19V DNA at low viremic concentrations here less than 10^6^ IU/mL (upper 95% confidence interval, 11.7%), only an anamnestic IgG response in one pre-transfusion seropositive recipient transfused with a highly viremic component containing greater than 10^10^ IU/mL B19V DNA (11). A later meta-analysis of 41 similar studies of B19V blood donor testing results showed that the prevalence of the B19V genome in blood donors was low (<1%), with corresponding low levels of B19V viremia, and a very low risk of transmission to blood recipients (10). More recent data from the United Kingdom showed 80 B19V-positive plasma pools (96 donations per pool) out of 76,065 donations screened, all with low B19V viral loads (17). In general, these findings suggest that B19V transmission via transfusion of blood components with low viremia levels is rare.

Even so, there is newfound concern about increased B19V respiratory transmission showing up in reactive pooled plasma specimens (18–21). During the first years of the COVID-19 pandemic, B19V was rarely found in pooled plasma testing, but several years later, following the break in the spread of B19V infection attributable to social distancing, test positivity for B19V increased sharply (33-fold in Europe, ~2-3-fold in the US) (22, 23). Public health authorities in the United States and Europe observed increased B19V activity in pooled plasma samples in 2024 (22, 24, 25); Canada did not observe marked increases in its nucleic acid testing for B19V (26). Specifically, in the United States, the percentage of plasma donors exceeding high viremia threshold (>10^4^ IU/mL) increased from 1.5% in late 2023 to 19.9% in June 2024 (25). Therefore, particularly during outbreak periods, targeted B19V screening may be something to consider to protect susceptible transfusion recipients.

Although this study was completed in 2020, recent research on the immune response to B19V highlights its ongoing relevance for blood supply safety in the post-pandemic era. Based on the 2024 risk assessment model proposed by the European Centre for Disease Prevention and Control, the majority of our US study population would be classified as low risk (subjects who received blood transfusions while they were on surgical protocols but were otherwise healthy) or moderate risk (immunosuppressed individuals, including transplant patients or patients with chronic hematopoietic diseases) (4). Even in immunocompetent individuals with sustained immune responses to B19V who are traditionally considered “low-risk,” some may not be fully protected against B19V infection or co-infections (27). Routine B19V DNA screening for individual blood donations is not a standard practice in the majority of countries because blood-borne B19V transmission is generally rare, but B19V transmission via blood transfusion cannot be ruled out and can be harmful for susceptible recipients, such as immunosuppressed individuals, individuals with chronic hematological diseases, or pregnant women. It seems this practice may be based on assumptions of low transmission risk rather than definitive exclusion of harm, suggesting an opportunity to reevaluate B19V surveillance and targeted screening strategies. Routine screening policy in non-outbreak periods could continue unchanged, and targeted risk-based approaches could be used during documented periods of increased donor viremia, integrating recent post-COVID-19 pandemic surveillance data into the decision framework for when to trigger intensified monitoring. Our findings included limited clinical and blood component data on the study subjects, but these data do not provide sufficient relevant evidence to propose a specific risk-based screening approach, as the samples were collected before the COVID-19 pandemic.

The study reported herein has several strengths. First, results come from a large biorepository of thousands of samples collected from a single epidemiologic study conducted over 20 years that link blood product donors and recipients. Many study participants were tested multiple times and/or are part of special populations, such as immunosuppressed persons who may be more susceptible to severe clinical outcomes from B19V infection; testing results from these vulnerable populations may better reflect the true risk of severe outcomes from a true positive transmission. In this cohort, where several study participants had diseases associated with immunodeficiency, no transfused patient had a recognized severe outcome related to parvovirus B19V, not even in the one previously reported case of confirmed B19V transmission, who had a history of multiple transfusions related to cancer surgery (12).

This study has several limitations related to cohort characteristics and diagnostic methods, but these reflect the long-term, prospective nature of the work. The DNA testing relied on an endpoint semi-quantitative PCR assay rather than quantitative PCR. As the initial objective of the study was to detect the presence or absence of B19 virus (viral DNA) in the study subjects, the nested PCR approach was considered to be sensitive enough and appropriate. Once qPCR became available to us to keep the testing protocol consistent throughout the long-term study, a qualitative detection of B19 DNA was prioritized over quantitation. As a result, we interpreted the results with caution, as differences in assay sensitivity may have led to underestimation of viral presence and limited the reliability of viral load comparisons across samples. Endpoint nested PCR may be less informative for quantitation and less sensitive to very low-level viremia than contemporary qPCR approaches, even as it ensured methodological continuity over two decades. The hospital-based cohort included a higher proportion of immunosuppressed recipients than the general blood donor population, which may limit generalizability to healthy blood donors or to B19V testing patterns in other geographic locations. The study closed enrollment at the beginning of the COVID-19 pandemic in 2020, and incomplete follow-up or specimen loss limited longitudinal analyses in some participants. Despite these limitations, the study provides a robust longitudinal dataset that offers important insights into B19V transmission risk and surveillance in transfusion settings. The assay detects genotypes 1 and 2 but not genotype 3 (which is mostly observed outside North America and is of less epidemiologic importance in the geographic area where we conducted this study) (28, 29). Finally, a subset of participants showed fluctuation in IgG status between 12 and 24 weeks or at the end of the study. Possible explanations for these findings include assay variability near the cutoff, sample handling effects, or changes in immunologic state in immunosuppressed recipients. Without additional clinical context, these findings do not constitute evidence of biological seroreversion or seroconversion. Reporting the distribution of index values for these fluctuating cases, rather than only positive or negative categories, could have made the interpretations more robust.

## Conclusion

These findings show a low incidence of B19V and a low transfusion-transmission risk of it over 20 years. Although the data may not provide enough evidence to consider revising current B19V testing strategies on their own, there are other sources of evidence from pooled plasma testing around the world suggesting that more widespread B19V testing of blood products could be indicated. Routine B19V DNA screening for individual blood donations is not a standard practice in the majority of the countries because blood-borne B19V transmission is generally rare, but even though rare, B19V transmission via blood transfusion cannot be ruled out and can also be particularly harmful for susceptible recipients, such as immunosuppressed individuals, individuals with chronic hematological diseases or pregnant women. We encourage the creation of more large prospective cohort studies to monitor for re-emerging pathogens such as B19V and newly emerging pathogens to maintain the safety of the blood supply.

## Data Availability

The raw data supporting the conclusions of this article will be made available by the authors, without undue reservation.

## Author disclaimer

This research was supported by the Intramural Research Program of the National Institutes of Health (NIH). The contributions of the NIH authors are considered Works of the United States Government. The findings and conclusions presented in this paper are those of the authors and do not necessarily reflect the views of the NIH or the U.S. Department of Health and Human Services.
